# High levels of serum glypican‐1 indicate poor prognosis in pancreatic ductal adenocarcinoma

**DOI:** 10.1002/cam4.1833

**Published:** 2018-10-24

**Authors:** Cong‐ya Zhou, Yi‐ping Dong, Xiao Sun, Xin Sui, Hong Zhu, Ya‐qin Zhao, Yuan‐yuan Zhang, Clifford Mason, Qing Zhu, Su‐xia Han

**Affiliations:** ^1^ Department of Oncology The First Affiliated Hospital, College of Medicine, Xi'an Jiaotong University Xi'an China; ^2^ Department of Abdominal Oncology West China Hospital of Sichuan University Chengdu China; ^3^ Department of Obstetrics and Gynecology University of Kansas School of Medicine Kansas City Kansas

**Keywords:** carbohydrate antigen 19‐9, diagnosis, glypican‐1, pancreatic ductal adenocarcinoma, prognosis, serum biomarker

## Abstract

Carbohydrate antigen 19‐9 (CA19‐9) fails to demonstrate the predictive value for early detection pancreatic ductal adenocarcinoma (PDAC). Glypican‐1 (GPC1+) exosomes may serve as a noninvasive diagnostic tool to detect early stages of PDAC. Therefore, it is necessary to explore the serum GPC1 levels and determine whether serum GPC1 serves as a novel biomarker for PDAC patients. Blood samples were collected from 156 patients with PDAC, 199 non‐cancer controls, and 240 patients with other cancers. Serological levels of GPC1 were examined by enzyme‐linked immunosorbent assay (ELISA). Finally, a 5‐year follow‐up was monitored to evaluate the correlation between serum GPC1 levels and overall survival in 156 patients with PDAC. The results suggested that levels of serum GPC1 and CA19‐9 were higher in PDAC patients than that of controls (*P* < 0.05). Serum GPC1 levels in PDAC were different from those in gallbladder carcinoma (*P* < 0.001), colorectal carcinoma (*P* < 0.001), gastric carcinoma (*P* < 0.001), and prostate cancer (*P* < 0.001), but not hepatocellular carcinoma (*P* = 0.395) and cholangiocarcinoma (*P* = 0.724). Receiver operating characteristic curve (ROC) analysis showed that serum CA19‐9 was significantly better than serum GPC1 in distinguishing PDAC patients from the controls (AUC, 95% CI: 0.908, 0.868‐0.947 vs 0.795, 0.749‐0.841, respectively). The serum GPC1 cannot be used as a serum diagnostic biomarker for PDAC patients. The level of serum GPC1 decreased 2 days after surgery (*P* = 0.001), which were not different from serum GPC1 levels in healthy control (*P* = 0.381). The overall survival rate was shorter in patients with high levels of serum GPC1 compared to those with low levels of serum GPC1 (log‐rank = 5.16, *P* = 0.023). Taken together, the results indicate that high levels of serum GPC1 predict poor prognosis in PDAC patients. Serum GPC1 may be a prognosis factor for PDAC patients.

## 1 INTRODUCTION

Pancreatic ductal adenocarcinoma (PDAC) is a malignant cancer and the fourth leading cause of cancer‐related deaths in Western countries.^1^ Because of the depth of anatomical location and the lack of specific symptoms at an early stage, PDAC is usually diagnosed at advanced stages when patients have metastasis and little chance of curative surgical therapy. The 5‐year survival rate of PDAC patients is only 7%.^2^, ^3^, ^4^ CA19‐9 is considered the gold‐standard and is routinely used for diagnosis of PDAC, but has limited utility as an early detection marker due to its variable sensitivity (60%‐90%) and specificity (68%‐91%).^5^ The levels of serum CA19‐9 also increase in other cancers, including extrahepatic bile duct cancer, gastric cancer, and colorectal cancer.^6^, ^7^, ^8^ Therefore, it is urgent to identify novel biomarkers to detect early‐stage PDAC to facilitate possible curative surgical therapy, and improve the prognosis and survival of PDAC patients.

Heparan sulfate proteoglycan glypican‐1 (GPC1) is a member of heparan sulfate proteoglycans (HSPG) family, which bind to the cell surface by a glycosylphosphatidylinositol anchor.^9^ Previous studies reported overexpression of GPC1 in pancreatic cancer and glioma and breast cancer, and suggested its involvement in tumor growth and angiogenesis.^10^, ^11^, ^12^ Overexpression of GPC1 also correlated with the poor prognosis and chemoresistance in esophageal squamous cell carcinoma.^13^ MiR‐96‐5p was shown to regulate GPC1 resulting in the inhibition of pancreatic cancer cell proliferation.^14^ Whipple and colleges found that GPC1 enhances tumor growth, angiogenesis, and invasion in Kras‐driven genetic mouse model of PDAC.^15^ Recently, Melo et al^16^ reported that tumor‐derived exosomes were enriched in GPC1, and GPC1+ exosomes were a biomarker for early PDAC detection. Therefore, GPC1+ exosomes may serve as a noninvasive diagnostic screening tool to detect early stages of PDAC. Melo et al only analyzed the levels of serum GPC1 in 56 patients with pancreatic cancer, and further studies with larger patient populations are needed to support their conclusions. The use of GPC1+ exosomes for diagnosing early‐stage PDAC is far from being clinically operative.^17^ In this study, we explored whether serum GPC1 levels could be used as a novel biomarker for detection of PDAC and whether serum levels of GPC1 can be as a prognostic factor for PDAC patients.

## 2 MATERIALS AND METHODS

### 2.1 Patient samples

**Figure 1:**
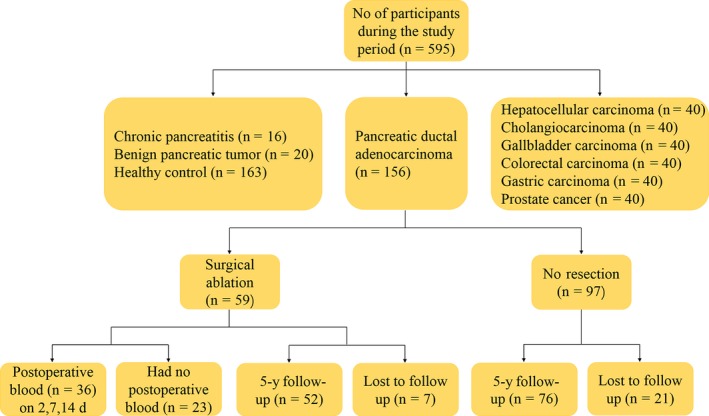
The process of this study

A total of 595 participants were eligible for this study. All participants’ blood samples were taken before treatment and collected between October 2011 and March 2017 from the First Affiliated Hospital of Xi'an Jiaotong University Medical College and the Tumor Hospital of Shaanxi Province (Xi'an, China). As shown in Figure 1, there were 156 patients with pancreatic cancer (histological verified PDAC), 199 controls which included 20 benign pancreatic tumor (BPT), 16 chronic pancreatitis (CP), and 163 healthy control (HC) patients. To test the specificity of serum GPC1 in cancers, we also collected serum samples from patients with hepatocellular carcinoma (n = 40), cholangiocarcinoma (n = 40), gallbladder carcinoma (n = 40), colorectal carcinoma (n = 40), gastric carcinoma (n = 40), and prostate cancer (n = 40). Furthermore, we followed 156 PDAC patients over a 5‐year period until all patients were deceased. Patients with PDAC were excluded if they had undergone radiotherapy or chemotherapy, had a previous history of cancer, or were suffering from acute pancreatitis when the study was initiated. All cancer patients were histological confirmed and diagnosed according to the American Joint Committee on Cancer staging (AJCC). All participants provided written informed consent.

### 2.2 Samples collection

For each participant, 5 mL of fasting blood was collected and centrifuged for 10 minutes at 1500 *g*. The serum was isolated, aliquoted into 3‐5 tubes, and stored at −80°C until analyzed. To determine serum GPC1 levels in preoperative blood, we analyzed the serum GPC1 levels of 36 PDAC patients on days 2, 7, and 14 after surgery. A follow‐up was implemented to evaluate the survival rate of PDAC patients 5 years’ post‐surgery.

### 2.3 ELISA

ELISA kits (RayBiotech, ELH‐GPC1, Norcross, GA, USA) were used to measure the levels of serum GPC1. Experiments were performed according to manufacturer's instructions. The optical density (OD) values were read at a wavelength of 450 nm using 96‐well microplate reader (Thermo Systems, Boston, MA, USA). The concentration of GPC1 was calculated from the standard curve. The levels of serum CA19‐9 were detected by automatic electrochemiluminescence detection.

### 2.4 Statistical analysis

SPSS version 18.0 (SPSS Inc., Chicago, IL, USA) was used for all statistical analyses. Nonparametric parameters were used to analyze the differences in expression levels of serum GPC1 and serum CA19‐9 between PDAC patients and controls, as well as in PDAC patients and patients with other cancer‐related diseases. The serum GPC1 levels were adjusted to account for differences in age and sex among the patients. Receiver operating characteristic curve (ROC) was used to evaluate the sensitivity, specificity, and respective areas under the curves (AUCs) with a 95% of confidence interval (CI) of GPC1 and CA19‐9. Survival curves were created by the Kaplan‐Meier method, and survival was compared using log‐rank tests.

## 3 RESULT

### 3.1 Participants’ characteristics

There were 156 PDAC patients and 199 non‐pancreatic cancer patients (HC, n = 163; CP, n = 16; BPT, n = 20). The average age was 60 (60.37) years old, 58.6% were men. Among 156 PDAC patients, the ages ranged from 36 to 84 years old, and the mean age was 62.7 years old. The PDAC patients were categorized according to AJCC stages into four groups which included stage I patients (n = 23), stage II patients (n = 49), stage III patients (n = 25), and stage IV patients (n = 59). Age, sex, and other clinical characteristics of the patients are shown in Table 1.

**Table 1:** Clinical characteristics of 355 participants

Variable	PC (N = 156)	CP (N = 16)	BPT (N = 20)	HC (N = 163)	*P* value
Age, N (%)					
>60	83 (53.2)	3 (18.8)	4 (20.0)	87 (53.4)	0.002
≤60	73 (46.8)	13 (81.2)	16 (80.0)	76 (46.6)	
Sex, N (%)					
Male	93 (59.6)	16 (100)	4 (20.0)	95 (58.3)	<0.001
Female	63 (40.4)	0 (0.0)	16 (80.0)	68 (41.7)	
Smoke, N (%)					
Yes	51 (32.7)	14 (87.5)	1 (5.0)		
No	105 (67.3)	2 (12.5)	19 (95.0)		
Drink, N (%)					
Yes	24 (15.4)	7 (43.8)	1 (5.0)		
No	132 (84.6)	9 (56.2)	19 (95.0)		
Diabetes, N (%)					
Yes	25 (16.0)	4 (25.0)	2 (10.0)		
No	131 (84.0)	12 (75.0)	18 (90.0)		
ABO blood type, N (%)					
O	34 (21.8)	4 (25.0)	9 (45.0)		
No‐O	114 (73.1)	11 (68.8)	11 (55.0)		
Missing	8 (5.1)	1 (6.2)	0 (0.0)		
Site, N (%)					
Head	107 (68.6)				
Neck	6 (3.8)				
Body	32 (20.5)				
Missing	11 (7.1)				
Tumor size, N (%)					
≤2 cm	12 (7.7)				
2‐5 cm	87 (55.8)				
>5 cm	39 (25.0)				
Missing	18 (11.5)				
TNM stage, N (%)					
I	23 (14.7)				
II	49 (31.4)				
III	25 (16.0)				
IV	59 (37.9)				

AJCC, American Joint Committee on Cancer staging; BPT, benign pancreatic tumor; CP, chronic pancreatitis; HC, healthy control; M, metastasis; N, node; PDAC, pancreatic ductal adenocarcinoma; TNM, T, tumor.

### 3.2 Serum level of CA19‐9 and GPC1 in PDAC patients and controls

**Figure 2:**
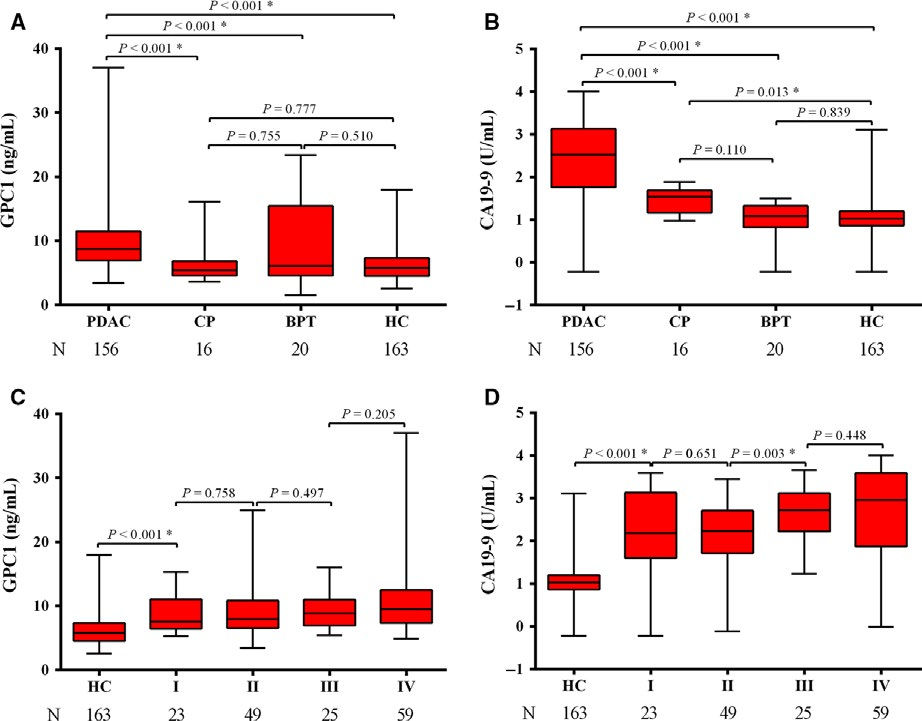
The serum levels of GPC1 and CA19‐9 in pancreatic diseases. The levels of serum GPC1 and CA19‐9 were determined by ELISA and automatic electrochemiluminescence. A, Serum GPC1 and B, serum CA19‐9 levels in various pancreatic diseases. Serum GPC1 levels were adjusted for differences in age and sex. C, Serum GPC1 and D, CA19‐9 levels in different stages of PDAC. Logarithmic function (log10) was used for the CA19‐9 (U/mL) *y*‐axis. * represent *P* value less than 0.05

As shown in Table 2, median serum GPC1 levels were significantly elevated in PDAC patients (8.75 ng/mL, interquartile range [IQR]: 6.92‐11.52) as compared with CP (5.44 ng/mL, IQR: 4.57‐6.83; *P* < 0.001), BPT (6.11 ng/mL, IQR: 4.57‐15.44; *P* < 0.001), and HC (5.78 ng/mL, IQR: 4.52‐7.37; *P* < 0.001) patients (Figure 2A). The serum GPC1 levels were higher in PDAC than that of HC patients. However, there was no difference in serum GPC1 levels in CP with BPT (*P* = 0.755), in BPT with HC (*P* = 0.510), and in CP with HC (*P* = 0.777). The levels of serum CA19‐9 (median, IQR) were significantly higher in PDAC than CP, BPT, and HC (334.95 U/mL, 57.62‐1344.75 U/mL vs 34.76 U/mL, 14.58‐48.79 U/mL, *P* < 0.001; 12.11 U/mL, 6.62‐21.33 U/mL, *P* < 0.001; 10.56 U/mL, 7.28‐15.75 U/mL, *P* < 0.001; Figure 2B). The levels of serum CA19‐9 were higher in CP compared with HC (*P* = 0.013). Higher serum levels of GPC1 and CA19‐9 were found in stage I PDAC samples compared with HC samples (*P* < 0.001; *P* < 0.001). There was no difference in serum GPC1 levels between stages I and II (*P* = 0.758), stages II and III (*P* = 0.497), and stages III and IV (*P* = 0.205; Figure 2C). The median levels of serum GPC1 were higher in stage IV than that of stages I‐III (9.52 vs 7.94; *P* = 0.007). This indicated that GPC1 might participate in PDAC metastasis.

**Table 2:** Serum GPC1 and CA19‐9 levels in 355 study participants and comparisons in PDAC and controls

Variable	Diagnosis	N	Median	Minimum	Q1	Q3	Maximum	*P* value
GPC1	HC	163	5.78	2.56	4.52	7.37	17.97	
	BPT	20	6.11	1.52	4.57	15.44	23.37	0.510^a^
	CP	16	5.44	3.61	4.57	6.83	16.13	0.777^a^, 0.755^b^
	PDAC	156	8.75	3.45	6.92	11.52	37.03	<0.001^a^, <0.001^b^, <0.001^c^
CA199	HC	163	10.56	0.60	7.28	15.75	1275.00	
	BPT	20	12.11	0.60	6.62	21.33	31.76	0.839^a^
	CP	16	34.76	9.39	14.58	48.79	76.77	0.013^a^, 0.110^b^
	PDAC	156	334.95	0.60	57.62	1344.75	10000.00	<0.001^a^, <0.001^b^, <0.001^c^

*P* value was analyzed when GPC1 was adjusted by age and sex.

Q1, first quartile; Q3, third quartile.

a Comparison vs healthy control (HC).

b Comparison vs benign pancreatic tumors (BPT).

c Comparison vs chronic pancreatitis (CP).

### 3.3 Serum level of GPC1 in PDAC patients and other diseases

**Figure 3:**
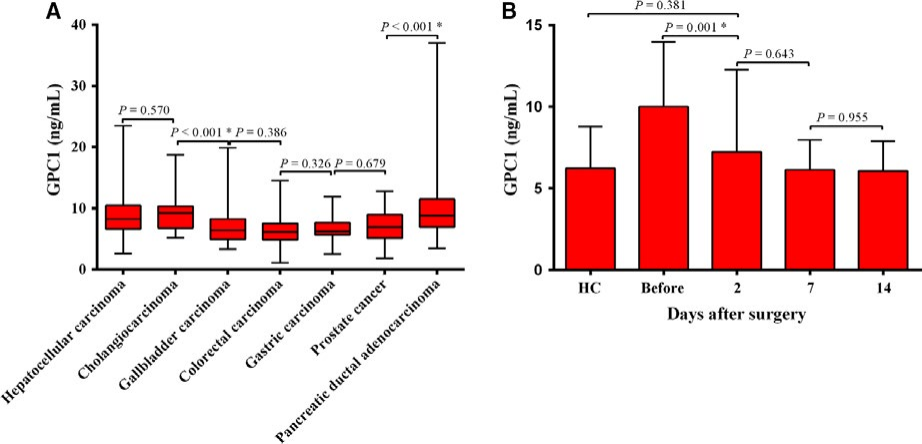
The serum levels of GPC1 in different diseases and in different days after surgery. A, Serum GPC1 levels in PDAC and other cancers. B, Serum GPC1 levels in the PDAC patients before surgery and 2, 7, 14 d after surgery. * represent *P* value less than 0.05

To explore the specificity of GPC1, we analyzed expression of GPC1 in hepatocellular carcinoma (n = 40), cholangiocarcinoma (n = 40), gallbladder carcinoma (n = 40), colorectal carcinoma (n = 40), gastric carcinoma (n = 40), and prostate cancer (n = 40). As shown in Figure 3A and Table S1, the levels of serum GPC1 were different in these diseases. For example, the serum GPC1 levels were higher in hepatocellular carcinoma (*P* < 0.001), cholangiocarcinoma (*P* < 0.001), gastric carcinoma (*P* = 0.019), and prostate cancer (*P* = 0.01) than in HC. The levels of serum GPC1 were not different among patients with HC and gallbladder carcinoma (*P* = 0.084) and colorectal carcinoma (*P* = 0.320). The levels of serum GPC1 could distinguish PDAC from gallbladder carcinoma (*P* < 0.001), colorectal carcinoma (*P* < 0.001), gastric carcinoma (*P* < 0.001), and prostate cancer (*P* < 0.001), but could not differentiate PDAC from hepatocellular carcinoma (*P* = 0.395) and cholangiocarcinoma (*P* = 0.724).

### 3.4 Serum GPC1 concentration before and after surgery

For 36 PDAC patients who underwent radical surgery, median serum GPC1 levels decreased significantly from 9.35 to 6.44 ng/mL (*P* = 0.001) at 2 days after surgery. And there was no difference compared to HC (median, 6.44 vs 5.78; *P* = 0.381; Figure 3B).

### 3.5 Diagnostic value of serum GPC1 and CA19‐9

**Figure 4:**
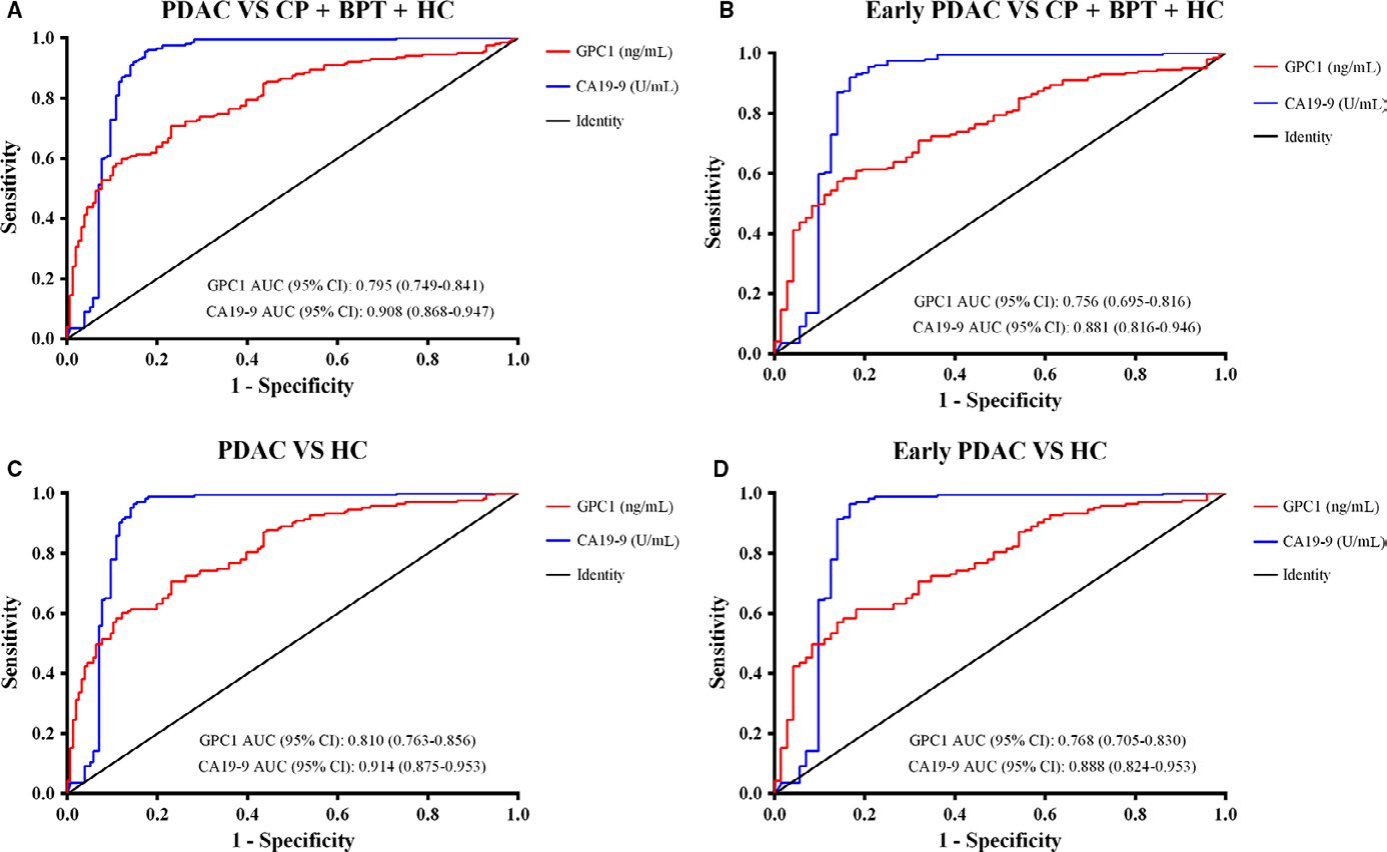
Receiver operating characteristic curve (ROC) analyses of serum GPC1 and CA19‐9 levels in the diagnosis of PDAC or early PDAC vs non‐malignant controls. A, ROC curve for GPC1 and CA19‐9 in PDAC patients vs disease controls. B, ROC curve for GPC1 and CA19‐9 in early PDAC patients vs disease controls. C, ROC curve for GPC1 and CA19‐9 in PDAC patients vs healthy controls. D, ROC curve for GPC1 and CA19‐9 in early PDAC patients vs healthy controls

Receiver operating characteristic curve curves were constructed between PDAC and controls. ROC curves showed the optimum diagnostic cutoff for GPC1 was 6.870 ng/mL and the optimum cutoff value for CA19‐9 was 37 U/mL when comparing PDAC with controls. Therefore, the cutoff of 6.870 ng/mL was selected to categorize PDAC patients with high or low serum GPC1 levels. The sensitivity, specificity, accuracy, and other indexes of GPC1 and CA19‐9 levels are shown in Table 3. The sensitivity and specificity of GPC1 (76.92% and 70.85%, respectively) were lower than that of CA19‐9 (82.69% and 93.97%, respectively) when comparing PDAC with the controls. The AUC for CA19‐9 was higher than that of GPC1 (0.908, 95% CI: 0.868‐0.947 vs 0.795, 95% CI: 0.749‐0.841; Figure 4A).

**Table 3:** Results for measurement of serum GPC1, CA19‐9, or both in the diagnosis of PDAC

	AUC	Sensitivity	Specificity	Accuracy	Youden's index	+PV	‐PV
PDAC vs HC+BPT+CP							
GPC1	0.795	76.92%	70.85%	73.52%	0.48	67.42%	81.36%
CA19‐9	0.908	82.69%	93.97%	89.01%	0.77	91.49%	87.38%
GPC1+CA19‐9		92.31%	65.83%	77.46%	0.58	67.92%	91.61%
PDAC vs HC							
GPC1	0.810	76.92%	70.55%	73.67%	0.47	71.43%	76.16%
CA19‐9	0.914	82.69%	97.55%	94.98%	0.80	97.30%	92.98%
GPC1+CA19‐9		92.31%	68.10%	79.94%	0.60	73.47%	90.24%
Early PDAC vs HC+BPT+CP							
GPC1	0.756	68.06%	70.85%	70.11%	0.39	45.79%	85.97%
CA19‐9	0.881	79.17%	93.97%	90.04%	0.73	82.61%	92.57%
GPC1+CA19‐9		93.06%	65.83%	73.06%	0.59	84.81%	68.23%
Early PDAC vs HC							
GPC1	0.768	68.06%	70.55%	69.79%	0.39	50.52%	83.33%
CA19‐9	0.888	79.17%	97.55%	91.91%	0.77	93.44%	91.38%
GPC1+CA19‐9		93.06%	68.10%	75.74%	0.61	56.30%	95.69%

The diagnostic cutoff values of serum GPC1 and CA19‐9 were 6.870 ng/mL and 37 U/mL, respectively.

AUC, area under the curve; BPT, benign pancreatic tumors; CA19‐9, carbohydrate antigen; CP, chronic pancreatitis; early PDAC, stage I and stage II; GPC1, glypican‐1; HC, healthy controls; PDAC, pancreatic ductal adenocarcinoma; +PV, positive predictive value; −PV, negative predictive value.

To test the hypothesis that the combination of GPC1 with CA19‐9 could improve the diagnostic value for PDAC, we analyzed the correlation between GPC1 and CA19‐9 both in the controls (CP, BPT, and HC) and in PDAC patients. The levels of serum GPC1 and CA19‐9 did not correlate with each other in the controls (correlation coefficient *r* = −0.057, *P* = 0.426), but had a mild positive correlation (correlation coefficient *r* = 0.188, *P* = 0.019) in PDAC patients.

When combining the AUC of CA19‐9 with GPC1, the sensitivity increased to 92.31%, which was higher than individual sensitivities. After excluding CP and BPT, the AUC for CA19‐9 (0.914, 95% CI: 0.875‐0.953) was greater than GPC1 (0.810, 95% CI: 0.763‐0.856) in PDAC compared to HC participants (Figure 4C). For early stage (stage I and stage II) of PDAC, the AUC of GPC1 was lower than that of CA19‐9 when compared to controls (0.756, 95% CI: 0.695‐0.816 vs 0.881, 95% CI: 0.816‐0.946; Figure 4B). The combination of sensitivity (93.06%) was also higher than the individual sensitivities. When comparing the early‐stage PDAC with HC participants, the AUC for CA19‐9 was higher than that of GPC1 (0.888, 95% CI: 0.824‐0.953 vs 0.768, 95% CI: 0.705‐0.830; Figure 4D), and the combined sensitivity increased to 93.06%. These data indicate that the combination of GPC1 with CA19‐9 could enhance the diagnostic value of CA19‐9 in PDAC patients.

### 3.6 Prognostic value of serum GPC1 levels for all PDAC and surgical PDAC patients

**Figure 5:**
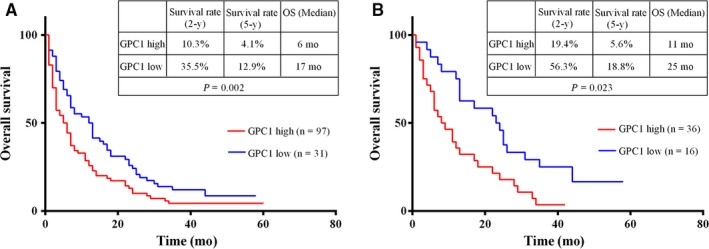
Kaplan‐Meier analysis of overall survival of PC patients. A, Kaplan‐Meier analysis of overall survival of PC patients (n = 128) with serum GPC1 level. B, Kaplan‐Meier analysis of overall survival of PC patients (n = 52) with surgical resection relative to preoperative serum GPC1 levels

For all 156 PDAC patients, at the end of the follow‐up period, 28 PDAC patients were lost. For the 128 PDAC patients, the 5‐year survival rate was 6.25%, and the overall median survival time was 7 months. The overall median survival time was 6 months for the group with higher GPC1 levels and 17 months for the group with lower GPC1 levels (log‐rank = 9.957, *P* = 0.002; Figure 5A). The 5‐year survival rate in the group with higher GPC1 levels was 4.1%. This was significantly different from 12.9% survival rate in the group with lower GPC1 levels. Next, we evaluated the relationship between the serum levels of GPC1 and the overall survival of PDAC patients. For those 52 PDAC patients who had undergone radical surgery, the overall median survival time was 13 months and the 5‐year survival rate was 9.6%. The overall median survival time was 11 months for the group with higher GPC1 levels and 25 months for the group with lower GPC1 levels (log‐rank = 5.16, *P* = 0.023; Figure 5B). These data indicated that the overall survival is shorter in the groups with high levels of serum GPC1 for both 128 PDAC patients (*P* = 0.002) and for 52 PDAC patients who had undergone radical surgery (*P* = 0.023). This suggested that high levels of serum GPC1 indicated poor prognosis in PDAC patients.

## 4 DISCUSSION

Pancreatic cancer is known for its low diagnosis rate at early stage and low 5‐year overall survival rate. The detection for early‐stage PDAC and the evaluation of PDAC prognosis remain difficult problems. Studies identified various proteins,^18^, ^19^ miRNAs,^20^, ^21^, ^22^ autoantibodies,^23^, ^24^ exosomes,^16^ metabolic biomarker,^25^ circulating tumor DNA, and circulating tumor cells^26^, ^27^ associated with PDAC. However, there are still no specific biomarkers for PDAC diagnosis at early stage. The identification of novel biomarkers is important for early‐stage PDAC detection and the prognosis and survival of PDAC patients.

In a previous study, we found that dickkopf‐1 was a diagnostic and prognostic biomarker for early‐stage pancreatic cancer.^28^ We also indicated that tumor‐associated antigens (TAAs) were supplementary serum biomarkers for the diagnosis of pancreatic cancer. In addition, Melo et al^16^ reported that GPC1+ exosomes could serve as a sensitive and specific biomarker for early PDAC detection. However, the use of GPC1+ exosomes as a diagnostic biomarker for PDAC is far from being clinically operative.^17^ In this study, we determined that serum GPC1 levels were higher in PDAC than in controls and found that the diagnostic value of serum GPC1 was inferior to CA19‐9. A major difference between this study and the study conducted by Melo et al^16^ is the number of patients recruited. In the former study, serum GPC1 levels were only determined in 56 PDAC patients, and the accuracy of the GPC1 sensitivity and specificity might be limited by their sample size. In our study, 159/163 (97.55%) serum CA19‐9 levels were within the normal range for HC participants, which could be another reason why the AUC of CA19‐9 was higher than GPC1. Although the sensitivity and specificity of serum GPC1 were lower than serum CA19‐9, the combined sensitivity (serum GPC1 and CA19‐9) was higher than each marker evaluated alone. However, the combined specificity decreased to <70% from 93% of CA19‐9 alone. As a result, the diagnostic accuracy in the combination of GPC1 and CA19‐9 was around 75%, which was much lower than the accuracy of CA19‐9 alone (around 90%), so the serum GPC1 was not useful for the detection of PDAC. Our results showed that there was a significant decrease in serum GPC1 levels 2 days after surgery (*P* < 0.001) which was not different with HC (*P* = 0.381). Recently, Duan et al and Lu reported that the expression of GPC1 was significantly higher in PDAC than in normal pancreatic tissues and that the higher GPC1 expression in PDAC tissues correlated to poor survival.^29^, ^30^ Whether serum GPC1 level was an independent prognosis factor in PC patients remains to be determined. In this study, we found that the overall survival was shorter in the patient group with high levels of serum GPC1 than in the group with low levels. Similar results were found for the 128 PDAC patients (*P* = 0.002) and the 52 PDAC patients who had underwent radical surgery (*P* = 0.023). The results suggest that high levels of serum GPC1 correspond to poor prognosis in PDAC patients.

Although CA19‐9 has some clinical significance for PDAC diagnosis, it is also elevated in other diseases such as hepatobiliary cancer, colorectal cancer, gastric cancer, and cholelithiasis. To verify the specificity of GPC1, patients with other diseases such as hepatocellular carcinoma, cholangiocarcinoma, gallbladder carcinoma, colorectal carcinoma, gastric carcinoma, and prostate cancer were evaluated. We found that serum levels of GPC1 can distinguish between PDAC and gallbladder carcinoma, colorectal carcinoma, gastric carcinoma, and prostate cancer, but could not differentiate PDAC from hepatocellular carcinoma and cholangiocarcinoma. We hypothesize that PDAC has similar biological behavior as hepatocellular carcinoma and cholangiocarcinoma, but not gallbladder carcinoma, colorectal carcinoma, gastric carcinoma, and prostate cancer. However, support for this supposition requires further investigation.

Based on our results, the median serum GPC1 levels in colorectal carcinoma were not different from that of healthy controls (6.10, IQR: 4.87‐7.53 vs 5.78, IQR: 4.52‐7.37; *P* = 0.320). This finding was different from that of De Robertis et.al^31^ who reported GPC1 overexpression in CRC tumor tissues. The possible reasons for this discrepancy could stem from the limited number of samples analyzed as well as the heterogeneity of the tissue and serum samples analyzed. These findings should be confirmed by further studies in a larger cohort.

Metastasis is the vital reason for death of PDAC patients. We found median serum GPC1 level was higher in PDAC patients who had metastasized (9.52 vs 7.94, *P* = 0.007). This suggests that GPC1 participates in metastasis of PDAC and is consistent with prior findings of GPC1 modulation of the angiogenic and metastatic potential of human and mouse cancer cells.^10^ The mechanism by which GPC1 alters metastasis of PDAC is unknown.

We acknowledge that there are limitations to the present study. For example, when comparing PDAC with all controls and HC, the specificity for CA19‐9 was 93.97% and 97.55%, which is higher than expected 68%‐91%.^5^ This could be due to the fact that 159/163 (97.55%) serum CA19‐9 level was within the normal range for HC participants. Meanwhile, Yuan previously reported that cigarette smoking was associated with a reduction in survival among patients with pancreatic cancer.^32^ In this study, we found that smoking indeed correlated with survival time in all 128 PDAC patients (*P* = 0.031), but not with survival time for those who had underwent radical surgery (*P* = 0.197). Collectively, it is plausible that the effect of radical surgery on survival covers the impact of smoking on survival.

In summary, our results indicate that the levels of serum GPC1 and serum CA19‐9 are higher in PDAC patients. And serum GPC1 levels decrease after surgery in PDAC patients. The combination of serum GPC1 and CA19‐9 increases the sensitivity of CA19‐9 in detecting PDAC. But the serum GPC1 cannot be used as a diagnostic biomarker for PDAC patients. Overall survival is also shorter in PDAC patients with high serum GPC1. We conclude that high level of serum GPC1 may be a prognostic factor for PDAC patients.

## CONFLICT OF INTEREST

The authors declare that they have no competing interests.
