# Supplementary material for: High levels of serum glypican‐1 indicate poor prognosis in pancreatic ductal adenocarcinoma
Source: Cancer Med. 2018 Oct 24;7(11):5525–33. doi: 10.1002/cam4.1833 (PMC6246926; doi:10.1002/cam4.1833)
Supplement: Supplementary file 1 [file CAM4-7-5525-s001.docx]

**Supporting table 1. The levels and comparisons of GPC1 (ng/ml) in different diseases**

| Diseases | N | Median | Minimum | Q1 | Q3 | Maximum | P value |
| --- | --- | --- | --- | --- | --- | --- | --- |
| Hepatic carcinoma | 40 | 8.25 | 2.62 | 6.64 | 10.46 | 23.51 | <0.001^a^, 0.395^b^ |
| Cholangiocarcinoma | 40 | 9.23 | 5.15 | 6.77 | 10.33 | 18.73 | <0.001^a^, 0.724^b^ |
| Gallbladder carcinoma | 40 | 6.41 | 3.31 | 4.94 | 8.28 | 19.89 | 0.084^a^, <0.001^b^ |
| Colorectal carcinoma | 40 | 6.10 | 1.08 | 4.87 | 7.53 | 14.52 | 0.320^a^, <0.001^b^ |
| Gastric carcinoma | 40 | 6.28 | 2.49 | 5.68 | 7.66 | 11.90 | 0.019^a^, <0.001^b^ |
| Prostate cancer | 40 | 6.90 | 1.87 | 5.10 | 8.96 | 12.80 | 0.01^a^, <0.001^b^ |
| Pancreatic cancer | 156 | 8.75 | 3.45 | 6.92 | 11.52 | 37.03 | <0.001^a^ |
| Healthy control | 163 | 5.78 | 2.56 | 4.52 | 7.37 | 17.97 | <0.001^b^ |

Q1, first quartile; Q3, third quartile. a, Comparison vs. HC; b, Comparison vs. PC.
